# Food Insecurity and Diabetes: The Role of Federally Qualified Health Centers as Pillars of Community Health

**DOI:** 10.7759/cureus.13841

**Published:** 2021-03-12

**Authors:** Anne Daly, Amit Sapra, Christine E Albers, Anastasia M Dufner, Priyanka Bhandari

**Affiliations:** 1 Department of Family and Community Medicine, Southern Illinois University School of Medicine, Springfield, USA

**Keywords:** food insecurity, food insecurity and diabetes, federal nutrition programs, role of federally qualified health centers, cycle of food insecurity and health, hunger, food insecurity and chronic disease, food assistance, feeding america, southern illinois university center for family medicine

## Abstract

Food insecurity is a complex public health problem affecting millions of people globally. It leads to negative health outcomes in the afflicted population and the society at large. There is a self-perpetuating vicious cycle between food insecurity and chronic health conditions like diabetes. It is important for healthcare professionals to be aware of its existence, to be able to recognize it, and to work with their patients to find solutions for it. Simultaneously, the providers should advocate for their patients and make program administrators, policymakers, and legislatures aware of this crisis. During the current coronavirus disease 2019 (COVID-19) pandemic, when economies have been badly affected and many people have lost their jobs, this subject has arguably assumed much greater importance. In this article, we discuss the magnitude of the problem, its relation to diabetes mellitus, and the role that a Federally Qualified Health Center (FQHC) can play in mitigating this problem.

## Introduction and background

In its most basic sense, food insecurity can be understood as a state where people lack the financial means to secure healthy foods. The United States Department of Agriculture (USDA) defines food insecurity as the inability to consistently obtain food without resorting to socially unacceptable practices [1]. The UN Health Hunger Report defines hunger as a period of severe food insecurity where individuals go for days without eating secondary to lack of finances, access, or other resources [2].

Food insecurity is a global health problem, and the glaring economic disparities between and within countries only make them worse. While it could be temporary or permanent, various political, social, economic, ethnic, racial, and financial determinants contribute to this complex issue. The USDA used an 18-item survey involving about 45,000 households during the Census Bureau’s Current Population Survey [Current Population Survey Food Security Supplement (CPS-FSS)]. Depending on the survey results, it divides households into high food secure, marginal food secure, low food secure, and very low food secure.

While one might think that developed countries such as the United States should be immune from the problem, this is far from true. As per the most recent USDA statistics of 2019, 13.6% of households with children in 2019 were affected by food insecurity in the US [3]. The situation has worsened during the ongoing coronavirus disease 2019 (COVID-19) pandemic, and as per the national projections, one in six people (more than 50 million individuals) and one in four children (17 million children) had been expected to be food-insecure in 2020. The projected annual food insecurity rates are at 15.6% (a 4.1% increase from 2018) [4].

People affected by food insecurity are at increased risk of various chronic health diseases, especially diabetes. Food insecurity often forces people to buy cheaper energy-dense food instead of healthy nutrient-rich food, leading to or worsening diabetes and its complications. Researchers state that about 9% of the US population is currently affected by type 2 diabetes mellitus, with a higher prevalence among African Americans, Hispanics, Native Americans, and Pima Indians [5]. Interestingly, these populations are also associated with higher rates of food insecurity [3].

## Review

Food insecurity and diabetes: the role of Federally Qualified Health Centers (FQHCs) as pillars of community health 

Food insecurity is widely recognized as a public health concern and a critical social determinant of health, given that healthy bodies and minds require access to nutritious meals throughout the life cycle. Food insecurity encompasses the physical sensation of hunger as well as compensatory behaviors used to avoid it. The compensatory behaviors related to food insecurity have enormous implications for preventing and managing chronic diseases. Individuals who worry that they will not have adequate money for food often reduce the variety of food intake and choose low-cost, energy-dense, and nutritionally deficient foods [6]. Other factors related to food insecurity include neighborhood food access, lack of transportation, and limited income, to name a few [7]. Food insecurity has been hypothesized as one mechanism by which poverty may predispose adults of low socioeconomic status to poor health outcomes.

According to the USDA, more than 35 million people in the US struggled with hunger in 2019 [8]. The rate is higher among some ethnic/racial minority groups, including African American and Latino populations, low-income households, and homes with a member who is disabled or homes headed by a single mother. The rate of food insecurity in individuals with diabetes may be as high as 20% [9]. In 2020, the COVID-19 pandemic massively affected the lives and livelihoods of people around the world. Millions of people are experiencing food insecurity for the first time, alongside those who have been experiencing food insecurity since before the pandemic began; the most vulnerable members of society tend to fare the worst. Unemployment rates have soared; workers in the service occupations, leisure, or hospitality industry have been especially hit hard. With the coronavirus pandemic's onset, more than 50 million people were estimated to experience food insecurity in 2020, including 17 million children [10]. A sharp increase in demand for charitable food assistance has been observed across the US, and this demand is expected to increase until the COVID-19 crisis is over. According to the Centers for Disease Control and Prevention, people with serious underlying medical conditions such as heart disease, diabetes, obesity, and lung disease, and older adults are at a higher risk of experiencing severe illness if they contract COVID-19. Cultivating an improved understanding of the role of food insecurity in diabetes management is now more urgent than ever to improve diabetes outcomes in vulnerable populations.

The cycle of food insecurity and health

**Figure 1:**
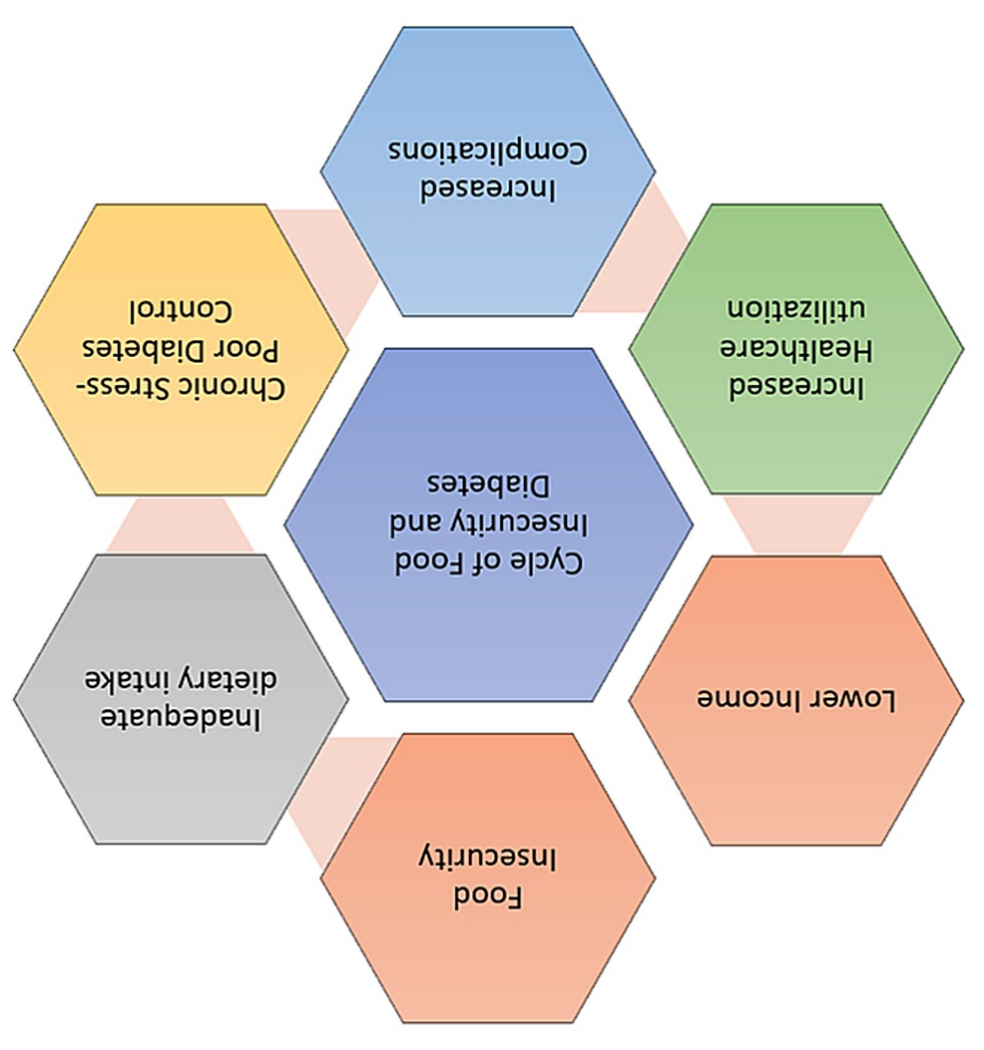
Cycle of food insecurity and chronic disease: diabetes Adapted from the article titled Hunger and Socioeconomic Disparities in Chronic Disease. New Engl J Med. 2010, 363:6-9 [6]

The intersection of hunger and health can be depicted as a vicious cycle (Figure 1).

The cycle includes alternating times per year of having an adequate food supply with periods of food scarcity. Food-insecure households are often forced to develop complex coping strategies such as visiting multiple stores to get the best deals [11] and shopping more often in stores carrying unhealthy foods, such as convenience stores and dollar stores [12]. These behaviors lead to purchasing cheaper foods that are high in calories but low in nutritional value.

Reliance on less healthy foods can lead to poor nutrition, weight gain, and increased risk for chronic diet-related diseases such as diabetes and obesity [13,14]. Limited knowledge, time, and resource scarcity contribute to the increased incidence of obesity [15]. These chronic illnesses can worsen existing disabilities or other chronic morbidities, resulting in higher healthcare costs [16] and difficulty in finding work [17]. These challenges further restrict the household food budget. Escaping this vicious cycle is extremely difficult once a person or family enters it. 

Food insecurity and diabetes

Chronic health conditions, including diabetes, hypertension, and obesity, are more prevalent in vulnerable populations compared to others. Household food insecurity increases the risk of being overweight or obese and increases the risk of diabetes by two to three times, which results in higher A1c values [18-21]. In a study of 711 participants, food-insecure individuals were found to have poor glycemic levels, defined as A1c of >8.5%, and an average A1c that is 0.47% higher compared to food-secure individuals; this was attributed to poor diet, poor medication adherence, poor self-management, and difficulty dealing with emotional stress. This survey is a valid measure of food insecurity as it demonstrates associations with reduced dietary variety, reduced intake of fruits, vegetables, and dairy products, increased consumption of calorically dense foods, and reduced micronutrient intake, all resulting in an increased incidence of obesity and diabetes [19].

Furthermore, the incidence of hyper-and hypoglycemia was higher in food-insecure individuals, further complicating diabetes management. Reasons for increased risk for hyperglycemia include the steady consumption of low-cost carbohydrate-rich processed foods, binge eating, inability to afford diabetes medications, and depression/anxiety/diabetes distress. Food-insecure individuals report reducing the amount of medication they take so that they have enough money to buy food [6]. These factors impair the patient’s ability to adhere to recommended diabetes daily self-care behaviors, worsen glycemic control, and increase the risk for diabetes-related complications. Frequent and severe hypoglycemia can occur due to inadequate or erratic carbohydrate consumption following sulfonylureas or insulin administration [22]. Food insecurity is an independent predictor of glycemic control, a relationship partially explained by difficulty following a recommended eating plan and increased emotional distress related to having diabetes [19]. Food insecurity may partly explain the observation that diabetes interventions are less effective among low-income populations [23].

Screening for food insecurity

A two-item screening tool has been developed and validated to assess the risk of food insecurity [24]. This tool includes the following statements: 1) “Within the past 12 months we worried whether our food would run out before we got money to buy more”, and 2) “Within the past 12 months the food we bought just didn’t last, and we didn’t have money to get more.” An affirmative response to either statement had a sensitivity of 97% and specificity of 83% [18].

Alternatively, the USDA Economic Research Service has developed 18-item, 10-item, and six-item shorter versions of food security questionnaires to help identify food-insecure households and households with very low food security with reasonably high specificity and sensitivity and minimal bias [25].

Federal nutrition programs and resources

Table 1 provides a summary of the various federal food nutrition programs and resources being offered in the US.

**Table 1:** Food nutrition resources and programs

Program	Service offered
Supplemental Nutrition Assistance Program (SNAP)	Nutritional assistance for low-income people and families
Special Supplemental Nutrition Program for Women, Infants, and Children (WIC)	Supplemental foods and nutrition education for low-income pregnant and postpartum women, infants, and children up to age five
Nutritional Lunch Program	Low-cost or free lunch for eligible children
School Breakfast Program	Low-cost or free breakfast for eligible children
Food banks	Free grocery items and meals
Meals on Wheels	Delivery of meals to seniors in need

**Figure 2:**
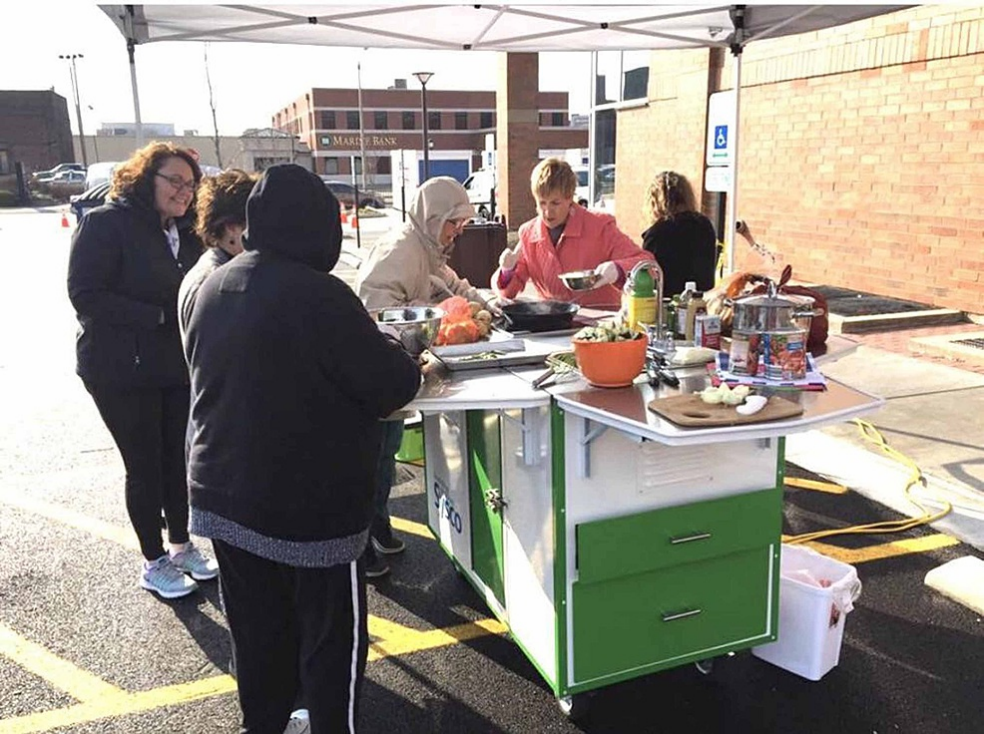
SIU Center for Family Medicine Diabetic educators teaching the public about healthy cooking during a health fair SIU: Southern Illinois University

Federal nutrition programs [26], such as the Supplemental Nutrition Assistance Program (SNAP) and the Special Supplemental Nutrition Program for Women, Infants, and Children (WIC), play a critical role in helping low-income families break out of the cycle of food insecurity and diet-related diseases. Both SNAP and WIC augment households’ food budgets, allowing them to purchase more healthy foods, and the Supplemental Nutrition Assistance Program - Education (SNAP-Ed) and WIC provide nutrition education to participants (Figure 2). Along with other federal nutrition programs that target specific populations, these programs make up the front line of the fight against food insecurity.

However, not everyone who is food-insecure qualifies for these federal programs; nationally, nearly three in 10 (29%) individuals who are estimated to be food-insecure and whose incomes are known live in households unlikely to qualify for more federal assistance [27]. Given the variation in food insecurity and state income and asset limits for specific programs, data indicate that the share of food-insecure individuals not eligible for public food assistance may be as high as 80% [28].

Feeding America: the nation’s leading domestic hunger-relief organization

The largest hunger-relief organization in the US is Feeding America. Through a network of 200 food banks and 60,000 food pantries and meal programs, Feeding America provides meals to 40 million people each year. 

Additionally, Feeding America conducts research and educates the public about the problem of hunger and advocates for legislation that protects people from going hungry. Feeding America’s research demonstrates that more than half of the households they serve have a family member with high blood pressure; one out of three households have at least one family member with diabetes; one out of five have an individual with a disability (i.e., hearing, visual, cognitive, ambulatory, self-care or independent living) [29]; and one out of four report poor or only fair health [30]. For 10 consecutive years, Feeding American has conducted Map the Meal Gap studies to understand and rectify the issue of food insecurity and food costs varying at local levels [31]. These studies provide a better understanding of how communities can develop more targeted strategies to reach people struggling with hunger.

In recent years, food banks have emerged as a potential partner in addressing traditional diabetes interventions in clinical settings. The primary expertise of food banks is in food distribution. The advantage food banks can provide lies in the fact that they already reach highly vulnerable populations, many of which are at the highest risk of poor engagement in traditional clinical settings.

With these trends in mind, Feeding America conducted a pilot study in 2014 to test the feasibility of providing diabetes self-management support and diabetes-appropriate food to diabetic individuals who rely on food banks. This pilot project was conducted at Feeding America food banks in three states; food banks provided diabetic people with diabetes education, healthy food boxes, and referrals to primary care providers. The pilot study findings were overwhelmingly positive; people with diabetes improved their blood glucose control, increased their consumption of healthy foods, and had better medication adherence. Significantly, the pilot project demonstrated that it is feasible to conduct health interventions like this in food bank settings [32].

Between 2015 and 2017, Feeding America launched a two-year randomized controlled research trial to demonstrate that food banks can improve food security and health outcomes for people facing food insecurity. The clinical trial named The Feeding America Intervention for Trial for Health-Diabetes Mellitus (FAITH-DM) enrolled food-pantry dependents living with type 2 diabetes at one of the following three food bank institutions: the Houston Food Bank, The Alameda County Community Food Bank, or the Gleaner’s Community Food Bank in Detroit. This study concluded that food banks could significantly improve food security and fruit and vegetable intake and food stability, besides reducing tradeoffs between food and diabetes management supplies. Study investigators did note the need to add in medication titration and educate people more intensively about carbohydrate intake and blood glucose control. The study also demonstrated that strong integration between food banks, healthcare, and other organizations addressing social determinants of health is needed and can enhance patient engagement and improved health outcomes and health disparities [23].

The role of FQHCs: stepping up to the plate to reach the medically underserved

The History of FQHCs

Federally Qualified Health Centers (FQHCs) are community-based health providers that receive funding from the Health Resources and Services Administration to serve as safety-net providers of primary care services for low-income people in underserved rural and urban areas [33,34]. The community health center model was developed as a federal demonstration program in 1965 as part of President Lyndon Johnson’s War on Poverty [35]. The term Federally Qualified Health Center was coined in 1989 [36]. In 2001, the number of FQHC sites jumped again as President George W. Bush launched a five-year expansion initiative. The Obama administration further developed the program with an infusion of funds through the American Recovery and Reinvestment Act of 2009 (ARRA) and the Patient Protection and Affordable Care Act (ACA) enacted in 2010. 

FQHC certification and services

To be certified as an FQHC, an entity must meet many requirements, including [34]:

· Serve an underserved area or population.

· Provide comprehensive services and have an ongoing quality assurance program including data reporting on a wide range of administrative and clinical requirements.

· Provide care on a sliding fee scale based on ability to pay.

· Operate under a governing board of directors composed mostly of FQHC patients.

· Complete annual reporting requirements.

· Provide access and integration for behavioral health, specialty care, and social services.

· Provide outpatient diabetes self-management training (DSMT) and medical nutrition therapy (MNT) for patients with diabetes or renal disease furnished by qualified providers of DSMT and MNT.

· Provide enabling services such as transportation, language interpretation, patient education, and/or case management. 

FQHCs are increasingly providing more supportive services, such as housing and food. Some FQHCs offer dental care, mental healthcare, substance use disorder treatment, on-site pharmacy services, and/or laboratory services.

The federal government endorses collaborations between FQHCs and other safety-net providers. FQHCs and their partners strive to reduce hospitalizations and patients’ episodic use of emergency departments (EDs) for non-urgent care needs, in order to control overall care costs. FQHCs have a long history of addressing and engaging with the social determinants of health as part of their core mission [37].

FQHCs lead by example to improve food insecurity: focus on Southern Illinois University Center for Family Medicine

Southern Illinois University Center for Family Medicine (SIU-CFM) was awarded FQHC status in 2012, following several years of planning and preparation. SIU-CFM had been serving a significant number of patients from vulnerable, underserved populations for many years. A recent community needs assessment has shown that 30-60% of the SIU-CFM patient population report food insecurity. Numerous efforts had been made to meet the growing needs and fill the gaps in these patients' clinical care. Over several years, a groundswell of community support for SIU-CFM to be made an FQHC had been building, including support from local and state health departments, many social service agencies, and both area hospitals. In 2012, the number of active FQHCs grew dramatically, with approximately 350 new sites being awarded the status nationally. SIU-CFM received word at that time that they were being awarded FQHC status. SIU-CFM was somewhat unique among FQHCs at the time, as it was affiliated with the state university system and an academic health institution housing a medical school, and it operates a well-recognized, highly prestigious family medicine residency training program for primary care providers specializing in family medicine. Becoming an FQHC enhanced SIU-CFM's status as an educational site, and it has emerged as a model setting for healthcare learners from all disciplines to gain exposure and experience with specialized training in socioeconomic determinants of health, providing expertise in understanding the issues germane to vulnerable populations, including food insecurity (Figure 2).

The Affordable Care Act (ACA) was signed into law in 2010 and was designed to provide affordable health insurance. Soon after its implementation, SIU-CFM encountered floods of new patients seeking care at our clinic who had not been receiving medical care previously. Once it attained the FQHC status, SIU-CFM was able to transform the level of access to services at our center. Clinic financial stability improved, primarily due to increased encounter rates and enhanced reimbursement rates. Types of benefits available to patients have expanded dramatically with the addition of multidisciplinary healthcare providers, including social workers, dietitians, diabetes educators, psychiatrists, community health workers, a full-time community resources specialist, and the addition of a medical-legal partnership. Patients gained access to the 340 B federal drug pricing program, which has been invaluable in enabling patients to afford medications, including insulins and other diabetes drugs.

**Figure 3:**
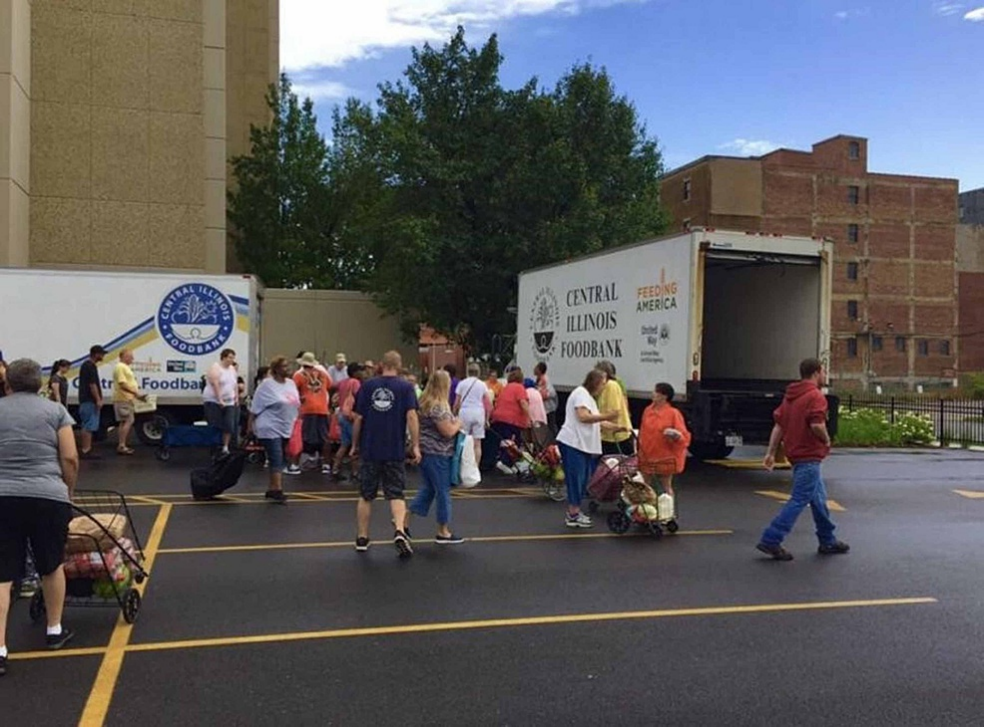
Central Illinois Foodbank distributing food at the FQHC at the SIU Center for Family Medicine FQHC: Federally Qualified Health Center; SIU: Southern Illinois University

Over time, expansion grants became available, and SIU-CFM was awarded funding for numerous initiatives. The SIU-CFM department chair had been an active member of the Board of Directors for the Central Illinois Foodbank, a regional member of the national Feeding America network. This experience led her to look for ways to improve the nutrient density of foods being distributed by the food drives and food pantries, including fresh foods, produce, protein, and dairy foods. Central Illinois Foodbank prepared a grant application to fund food distribution drives of fresh foods on-site at the SIU-CFM location (Figure 3). With this grant's approval, SIU-CFM began providing quarterly on-site food distributions of exclusively fresh foods, such as dairy products, lean protein, fresh fruits, and vegetables. During the first year, SIU-CFM distributed 1,000 pounds of fresh foods per week; in the second year, 2,000 pounds of fresh foods per week were distributed from the clinic's front lobby area.

**Figure 4:**
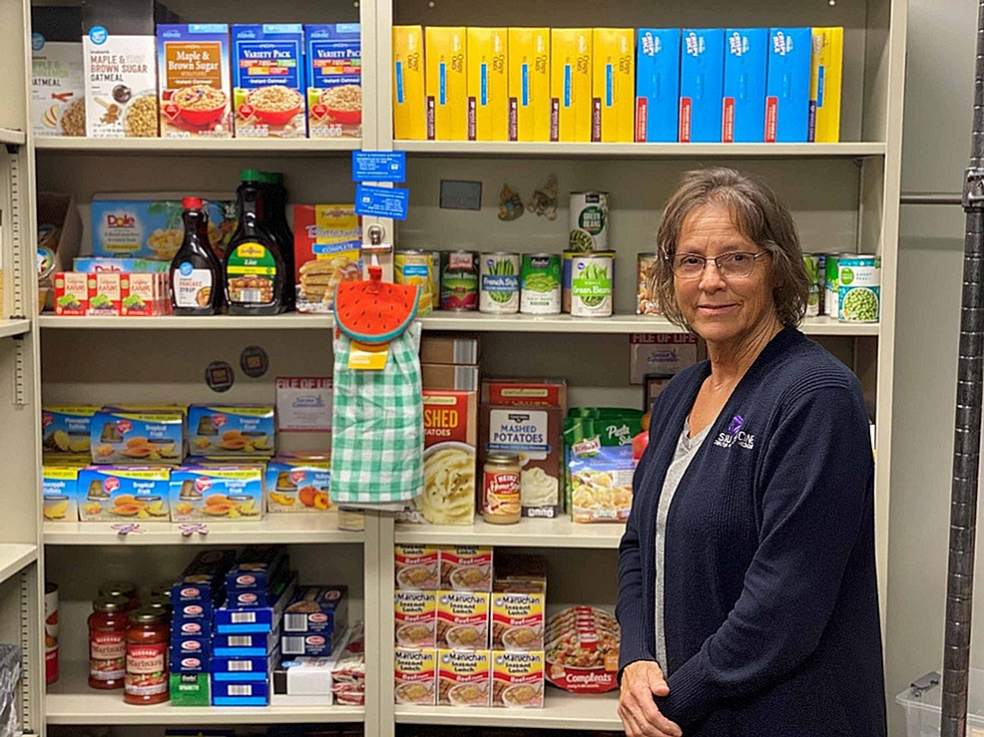
SIU Center for Family Medicine (FQHC) food pantry SIU: Southern Illinois University; FQHC: Federally Qualified Health Center

**Figure 5:**
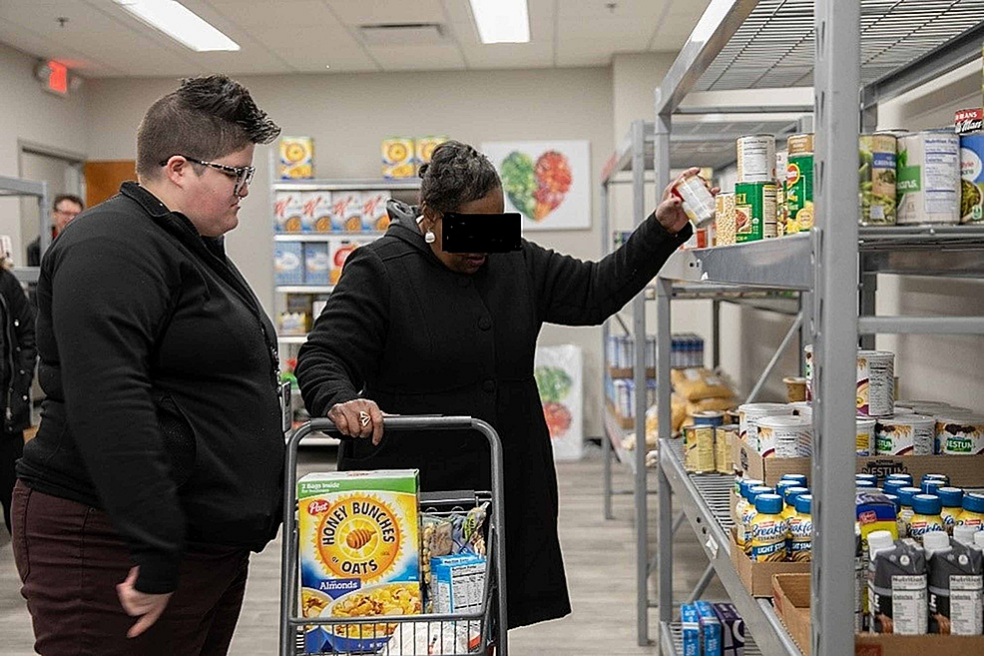
A patient getting food and other supplies at the SIU Center for Family Medicine (FQHC) food pantry SIU: Southern Illinois University; FQHC: Federally Qualified Health Center

Funding was acquired to purchase large refrigerators to store foods overnight; clinic staff was assigned to manage the setup and takedown of foods daily. Central Illinois Foodbank received support from a local business to purchase a mobile kitchen cart, which allowed hands-on cooking demonstrations, including food sampling of recipes and recipe distribution. Also, SIU-CFM maintains an on-site food pantry of non-perishable foods donated by clinic staff and other SIU School of Medicine employees (Figures 4, 5).

All healthcare entities, regardless of their status as FQHC providers, can take an active role in improving food security. One example is West Suburban Hospital in the Chicago area. While not an FQHC site, West Suburban Hospital has developed a close partnership with the Greater Chicago Food Depository and other community organizations to develop a model program designed to improve food insecurity. They fully integrate two validated food insecurity questions into their electronic health record. Participants who screen positive are given the option to join the Eat and Be Well program, which offers two dates per month to pick up fresh foods only at a designated location, including lean protein, dairy products, and fresh fruits and vegetables. Subsequently, qualitative and quantitative research was conducted, comparing individuals with and without diabetes who used the healthy food pantry connected to their hospital, with fresh foods only being distributed for six months, versus those who used other local pantries for six months. Investigators studied changes in A1c, blood pressure, and BMI. While the study's size and duration were limited, data did show people who had access to healthy food pantry services reported improved quality of life, particularly lower stress levels.

Suggestions of specific strategies that all healthcare facilities could consider adding at their worksite

* Develop process/procedure to integrate food insecurity screening questions to clinical practice, including adding to electronic medical record template.

* Build partnerships with community resources, including charities, churches, food banks, governments, and the private sector.

* Maintain/disseminate updated lists of community resources with contact information for services such as addiction treatment, child care, counseling, disability services, domestic violence, educational resources, emergency shelters, food programs, housing, clothing and furniture, medical assistance, legal assistance, senior services, transportation providers, assistance in registering or SNAP and WIC, and help in signing up for health insurance.

* Establish an on-site food pantry with non-perishable foods for those with emergency needs.

* Provide lists of recommended food donations to food pantries, with emphasis on nutrient density, including items for persons with modified dietary needs.

Call to action: how healthcare professionals can make a difference in improving food insecurity

Hunger is a national crisis that affects people from all walks of life. All Americans have a neighbor, a child's classmate, or co-worker who may be struggling to get enough food to eat. Healthcare providers work on the front lines to help get food in the hands of the people who need it the most. A recent consensus statement by major associations, including the American Diabetes Association, Association of Diabetes Care & Education Specialists, Academy of Nutrition and Dietetics, American Academy of Family Physicians, American Academy of Physician Assistants, American Association of Nurse Practitioners, and American Pharmacists Association, recommend that diabetes care providers expand awareness, access, as well as utilization of innovative and nontraditional diabetes self-management education and support. Understanding the root causes of hunger and food insecurity is one way to acquire the potential to demonstrate improved health outcomes and quality of life in a cost-effective manner. Since the outbreak of the coronavirus pandemic, food insecurity has exploded across the country. Gaining a deeper understanding of structural and systemic inequalities that disproportionately impact vulnerable populations is very much the need of the hour. Active partnerships between the vast network of charities, food banks, governments, and the private sector are needed to help fight hunger. Following are some of the initiatives that can be taken up:

* Advocate for the use of SNAP and LINK cards to be used at community farmer's markets.

* Promote community gardens to teach gardening skills, and how to harvest and use produce in food preparation.

* Establish methods to offer discounted rates/gift cards at local grocery outlets.

* Offer cooking classes/demonstrations on how to prepare healthy foods, including cooking on a budget.

## Conclusions

In this review article, we discussed in detail how the issue of food insecurity and associated healthcare problems such as diabetes have seriously affected the health and livelihoods of millions of people in the US, especially since the onset of the COVID-19 pandemic. Entities like Feeding America and FQHCs are doing an excellent job to tackle this crisis by providing various services to help those in need. FQHCs, in particular, have lived up to their reputation as the pillars of community health. SIU-CFM was awarded the FQHC status in 2012, and the organization has been at the forefront of fighting food insecurity by offering its valuable services such as a well-equipped food pantry and various healthcare-related assistance programs, especially to people from vulnerable and underserved communities. We also provided a brief overview of further actions/initiatives that healthcare professionals involved in the fight against food insecurity can engage in.
